# Techno-Economic Analysis of Vacuum Membrane Distillation for Seawater Desalination

**DOI:** 10.3390/membranes13030339

**Published:** 2023-03-15

**Authors:** Hassaan Idrees, Sara Ali, Muhammad Sajid, Muhammad Rashid, Fahad Iqbal Khawaja, Zaib Ali, Muhammad Nabeel Anwar

**Affiliations:** 1 School of Mechanical and Manufacturing Engineering (SMME), National University of Sciences and Technology (NUST), Islamabad 44000, Pakistan; 2 Artificial Intelligence for Mechanical Systems (AIMS) Lab, School of Interdisciplinary Engineering and Sciences (SINES), National University of Sciences and Technology (NUST), Islamabad 44000, Pakistan; 3 Human Robot Interaction (HRI) Lab, School of Interdisciplinary Engineering and Sciences (SINES), National University of Sciences and Technology (NUST), Islamabad 44000, Pakistan; 4 Intelligent Field Robotics Lab (IFRL), National Center for Artificial Intelligence (NCAI), National University of Sciences and Technology (NUST), Islamabad 44000, Pakistan; 5 Department of Computer Science, National University of Technology (NUTECH), Islamabad 44000, Pakistan

**Keywords:** membrane desalination, vacuum membrane desalination, techno-economic analysis

## Abstract

Seawater desalination is an affordable and viable solution to the growing freshwater scarcity problem in water scarce regions. The current study focuses on cost analysis of Vacuum Membrane Distillation (VMD) setup for removing salts from water. The membrane used in the flat sheet VMD module was Polytetrafluoroethylene (PTFE) with 250 mm × 200 mm dimensions and 165 µm thickness. The experiments were carried out with variations in parameters such as velocity, pressure, concentration, and temperature. For the cost analysis, the operational, maintenance, instrumentation, and capital cost of the lab model was considered and then upscaled. A range of experiments was performed for NaCl and KCl under variations of operating parameters. It was noted that, for the NaCl solution, the increase in temperature from 50 °C to 70 °C doubled the permeate flux. However, for the conditions tested, the concentration shift from 0.25 M to 0.75 M decreased the permeate flux by 1.4% because the increase in ion concentrations along the membrane lowers the vapor pressure, restricting the permeate flux. The results trend for the KCl solution was similar to the NaCl; at temperature T1, it was noted that increased concentration from 0.25 M to 0.75 M significantly reduces the permeate flow. The reduction in permeate flow was nonlinear for a given pressure 30 kPa and velocity 5.22 m/s, but linear for all other variables. It was also observed that with an increase in temperature from 60 °C to 70 °C, the permeate flux for concentration 0.25 M was 49% for all the combinations of pressure and velocity. In addition, permeate flow increased 53% from temperature 50 °C to 60 °C and 49% from temperature 60 °C to 70 °C for both the solutions at a concentration of 0.25 M. This shows that the temperature also had a profound impact on the permeate flux. The economic analysis and market survey shows that the cost of clean water at the lab level was high which can be significantly reduced using a large-scale setup providing 1,000,000 L/H of distilled water.

## 1. Introduction

A country’s growth depends on clean water resources and their proper management, as it is the most essential requirement for agricultural and industrial sectors. Once an abundant resource, pollution, population growth, and climate change have depleted fresh water supplies. According to a survey by Water Aid Organization, almost 663 million people on earth are lacking clean water [1]. Numerous desalination techniques are used to remove salts from sea water, such as Reverse Osmosis (RO) [2], multi-effect distillation (MED) [3], Multistage Flash Distillation (MFD) [4], and Membrane Distillation (MD). Another desalination technique is the use of a vacuum heat pump, explored by Bin et al. [5]. According to the World Health Organization (WHO) the goal of desalination is to reach a salinity of less than 500 mg/L for drinking water [6,7].

With the constant advancement of membrane separation technology, its applicability is rapidly growing. Membrane filtration technology is currently applicable in various industrial fields for serving various purposes such as in high performance chemical reactive clothing [8], laser ignition [9], and desalination of seawater [10]. Based on the techniques used to create a vapor pressure gradient across the membrane and collect the transported vapors from the permeate side, the MD processes are categorized into four basic configurations [7] which have crucial separation performance and operating costs [11,12].

**Figure 1:**
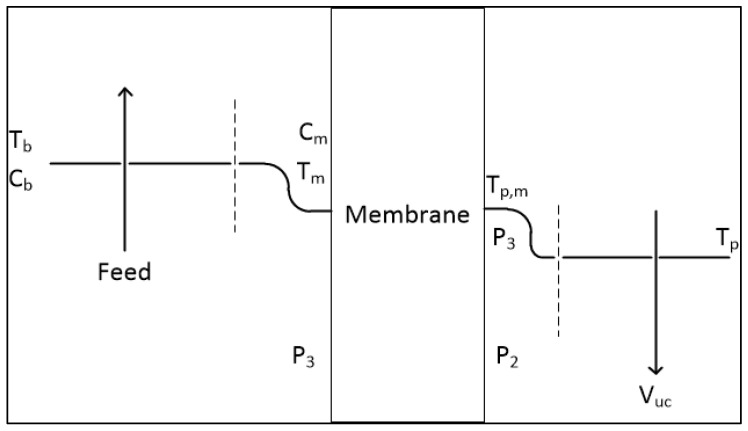
Subsystems in VMD Process.

**Figure 2:**
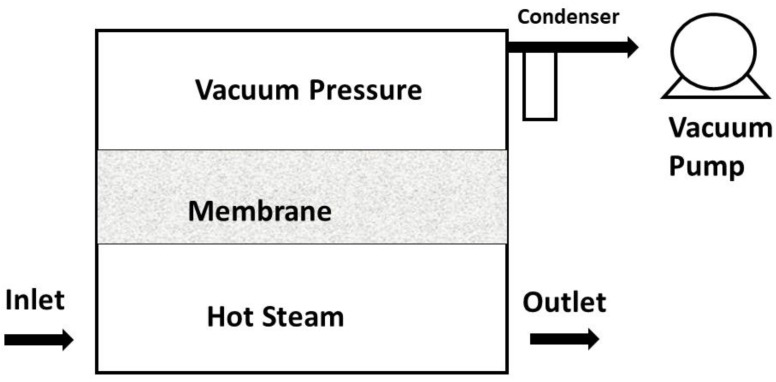
Vacuum Membrane Distillation Process.

Among the types of MD processes discussed, VMD is considered to be an appealing and cost-effective membrane separation process [13]. It provides a higher-pressure gradient than other MD methods, with low operating pressures [14]. One side of the membrane is vacuumed, and the other side is kept slightly below the saturation pressure, which helps in the filtration process [15,16]. In a VMD process, heat and mass transfer processes occur concurrently. The system consists of the bulk feed solution, the feed boundary layer (next to the membrane), the membrane, permeate boundary layer, and the permeate bulk area as indicated in Figure 1, and the general process diagram of VMD is shown in Figure 2.

Due to the existence of a vacuum, the boundary layer is thinner close to the membrane on the permeate side. This lack of a boundary layer allows VMD to have a greater 70 flux than DCMD, AGMD, and SGMD. This study will focus on using VMD technology to desalinate seawater because of the advantage offered by a thinner boundary layer leading to greater permeate flux. Compared to other methods, it provides several distinct benefits, including chemical-free operation, usability, environmental friendliness, and a well-understood process [7].

The cost of setting up a membrane distillation unit has been the subject of several studies. Exergy analysis, sensitivity studies, and economic analysis were performed by Al-Obaidani et al. [17]. The focus of the research was to determine the viability of the DCMD procedure. Water product costs for DCMD with heat recovery employing an MD system were projected to be $1.17/m^3^ (24,000 m^3^/day). Banat and Jwaied [18] calculated the small unit’s ($15/m^3^) and big unit’s ($18/m^3^) costs for producing potable water. Under the ideal circumstances, the cost of producing 5 m^3^ of water per day with the MD system (ignoring the price of the waste heat used) may be less than $1, as shown by Ali et al. [19]. Using a power plant (36.6 m^3^/day), Kullab and Martin [20] calculated the cost of product water as $1.13/m^3^. Using an air gap membrane distillation module (105,000 m^3^/day), Meindersma et al. [21] calculated the cost of product water as $0.26/m^3^. Sarbatly and Chiam [22] calculated that using geothermal energy in desalination using vacuum membrane distillation may save at least $0.72/m^3^ for the distilled water. The use of low-grade waste heat or other energy resources like wind, solar, and geothermal energy to lower the price of water products has also been the subject of research among the researchers [23,24]. For industrial scale plants, the aim of the researchers is to develop an energy efficient process that aligns with its production capabilities. In this context, Dong et al. [25] developed open-source simulators to predict MD performances; results were aligned with the laboratory results. The use of these open-source simulators is a critical step in the process of commercialization of MD and VMD plants. A laboratory-scale experiment was conducted on a polyvinylidene fluoride hollow-fiber vacuum membrane distillation (VMD) module before constructing a VMD seawater desalination demonstration plant with a capacity of 400 m^3^/d. The hollow-fiber VMD module was tested under various feedwater conditions, and the results showed high permeate flux and salt rejection rates [26]. The memsys V-MEMD module [27], which uses hydrophobic membranes and vacuum to enhance the membrane distillation process, has advantages in lower investment and operational cost and higher energy efficiency. A solar-driven memsys system illustrated good operating performance, while a diesel heater-driven system investigated the effects of heating, cooling, and feed conditions on module performance. The memsys module has great potential for the industrialization of MD technology. A consortium [28] evaluated the application of MD for the desalination of concentrated brines from thermal plants. Two MD technologies based on the multi-effect vacuum and air gap were tested at a full-scale thermal desalination plant in Qatar, and both showed high salt rejection rates and stable flux, demonstrating the feasibility of MD to produce potable quality water from brines discharged from thermal desalination plants.

This study focuses on the cost analysis of VMD in removing salts (NaCl and KCl) from seawater under various operating conditions. The reason why KCl was used in our study is the abundance of KCl salts in the seawater [29,30]. The study also takes into account the effect of different parameters such as velocity, pressure, and temperature on permeate flux. In addition to previous literature the study is based on technoeconomic analysis of VMD in removal of NaCl and KCl from seawater under various operating conditions. The cost of a lab scale VMD setup was calculated and then projected to an industrial scale plant at breakeven point for 10,000 L/H production of distilled water.

## 2. Methodology

### 2.1. Governing Equations

The permeate flux in the VMD can be studied by the Knudsen diffusion model [20].

(1) $$J=A*\;\Delta P=A*\left(P_{m}\;\;\left(T_{m},\;x_{salt}\right)-P_{v}\right)$$

where J is the permeate flux, A is the MD coefficient, ΔP is the pressure difference across the membrane, $P_{m}$ and $P_{v}$ are the vapor pressure on the feed and vacuum side, respectively, $T_{m}$ is the temperature of feed adjacent to the membrane, and $x_{salt}$ is the mole fraction of the salt in the solution.

The MD coefficient A, is given by [23,31]:

(2) $$A=\;\;1.064*\;\frac{p*\;\varepsilon }{m*\;t}*{\left(\frac{N}{R*T}\right)}^{\frac{1}{2}}$$

where p is the membrane pore size, ε is the porosity, m is the membrane thickness, t is the tortuosity, R is the gas constant, and N is the molecular mass of water. The permeate flux has been measured experimentally in this work for all the cases as shown in Section 3.1.

### 2.2. Experimental Setup

**Figure 3:**
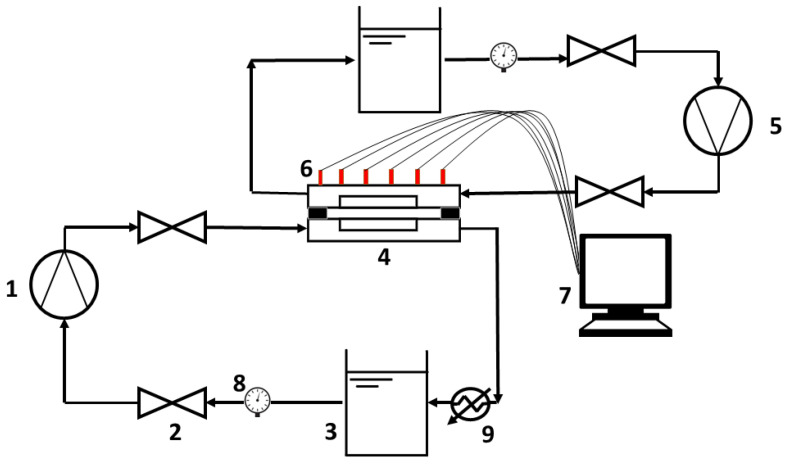
Schematic for experimental setup: 1. Feedwater Pump, 2. Valve, 3. Feedwater Valve, 4. VMD Module, 5. Vacuum Pump, 6. Pressure Sensors, 7. Computer, 8. Flow Gauge, 9. Heater.

**Figure 4:**
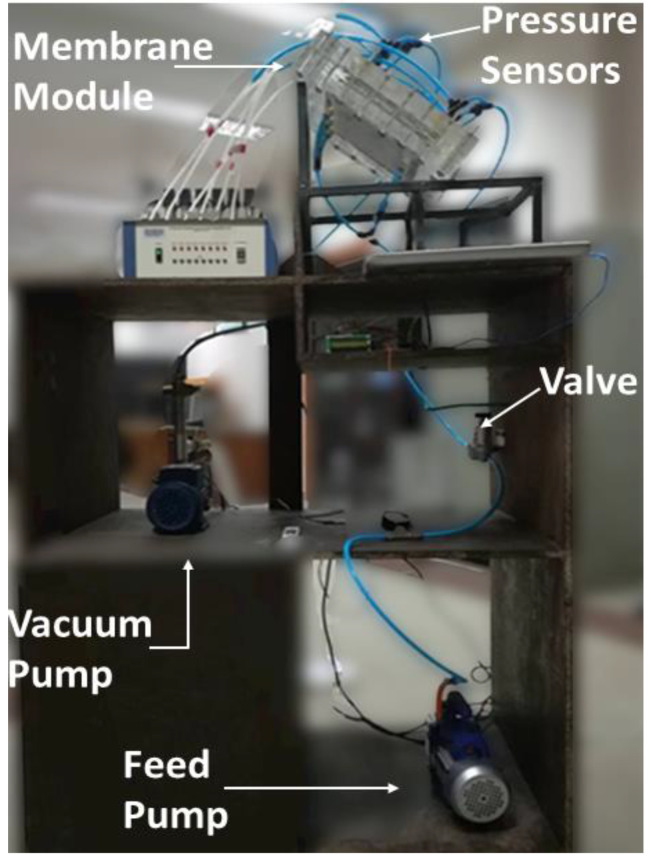
Experimental Bench for VMD.

The experimental setup consists of two pumps, one feedwater, one vacuum pump (Hyundai Vacuum Pump 0.5 Hp, HCPSP0.5-1 × 1IN), a VMD membrane module, Control Valves, Pressure sensors, and water storage tanks. The details of these are given in Figure 3 and Figure 4. The VMD module in Figure 3 is connected to a computer for data acquisition, where pressure data is being communicated from multiple probes as indicated between module 4 and 7 in the figure.

The membrane used in the DCMD module was a Polytetrafluoroethylene (PTFE) flat sheet of hydrophobic membrane purchased from commercial supplier Sterlitech. PTFE is one of the primary membranes used for desalination processes [32] and is suitable for the current study as it has shown promising results at the current operating temperatures and flux. The dimensional details are given in Table 1 below.

The feedwater used for filtration was prepared by dissolving NaCl and KCl salts in Type 1 distilled water. Both salts were procured from Sigma-Aldrich with maximum impurity of 0.0001% that meets analytical specification of Ph, Eur., BP., USP, FCC, E508. An Arduino based TDS meter from MT Technology Co., Ltd. (Tokyo, Japan) was used for measuring the salt content in real-time for the solution. The concentration of salts was determined by taking samples and measuring their conductivity using the conductivity meter. The values were plotted on the graph against the standard curve between the concentration and conductivity for the sample NaCl and KCl salts, and the values are reported in the molar concentration units. The absolute pressures at the feed channel’s six inlets and outlets were measured using a pressure bench. The six different pressure measurements are there to obtain pressure values at six different points. This helps to address the non-uniformity of pressure distribution at various points within the membrane. Although the uniformities are small thereby at the end, an average value was taken for pressure which was used for our experiments. The pressure bench was bought from Electrical Engineering Services Pakistan (model FM 1849-19A), with a range of 0–6 bar and 8 pressure channels.

Experiments were conducted at various temperatures, feed flow rates, and salt concentrations. In addition, a vacuum regulator was employed to regulate the vacuum pressure in the permeate chamber, and a study of permeate pressure variation was also done. Table 2 below lists experimental operating settings and several sets of experiments. The operating parameters used were Concentration (C), Pressure (P), Velocity (V), and Temperature (T) with variations given in Table 2, and the range of control parameters was decided based on actual conditions.

The flow rates for the experimentations are between 3.48 and 5.22 m/s. This higher flowrate was chosen to achieve optimal salt removal and the lowest heat losses. The flow was controlled using a globe valve linked to the water pump and monitored with an Arduino-connected flow meter. The permeate side vapors are condensed into water droplets in an external condenser. The water droplets are sent from the condenser to a beaker. The process is allowed to evolve until the system’s output becomes consistent. After the process has been wholly matured, the rise in the beaker’s water level is measured over a period for which the beaker’s water was left to settle. The permeate flux (J) has a unit of L/M^2^_._H.

The temperature of the feed reservoir is recorded using a water-resistant DS18B20 sensor. Arduino is used for the recording of temperature data. The information is then utilized to examine the behavior of the transmembrane flow at various temperatures. The temperature data are also used to adjust the temperature of the reservoir. An Arduino controls the relay, which in turn controls the heating coil. Arduino gets a signal from the temperature sensor when the specified temperature is attained. It then deactivates the relay to halt the heating operation until the temperature of the reservoir falls below the necessary level.

### 2.3. Economic Analysis

The cost of desalination discussed in this paper is based on capital, operation and maintenance, installation, and human resource costs. The capital expenses and operating expenses were estimated for the up-scaled model using the equipment factor estimate method [33,34]. The estimate was generated by taking the operational and capital cost and then adding the human resource and miscellaneous cost factors. The calculations were carried out for a range of investments from lab scale setup with 1 L/h capacity to an upscaled industrial model of 1,000,000 L/h capacity. The important cost analysis parameter included was the breakeven point for the proposed model in comparison with commercially available bottled water. A big percentage of the cost of desalination setups consisted of the operating costs which included the electrical and maintenance cost of the pumps. The pumps capital and operating costs for different proposed scales of VMD plants were calculated with the help of local and globally available pumps. The market survey was conducted by contacting local vendors and suppliers who provided quotations for price estimation with a validity of 30 days as per standard procurement practice. The human resource and miscellaneous costs were different for lab and industrial setups, with percentage of human resource cost for lab scale greater than that of industrial level while miscellaneous costs greater in case of an upscaled model.

The focus of techno economic analysis is to provide the starting point for potential investors in VMD distillation. Some of the challenges in moving from lab scale to industrial scale of VMD process include high energy consumption, fouling, scaling, and pore wetting [35]. The deviations in cost estimation will be dependent on diverse socio-economic factors and prevailing conditions. This study, therefore, serves to provide an indicative cost to potential investors for upscaling cost from lab to industrial scale.

## 3. Results

In view of the parameters discussed in Table 2, a total of 72 experiments were conducted, with 36 each for NaCl and KCl. The experiments were conducted over the variation of Temperature, Concentration, Velocity, and Pressure.

### 3.1. Experimental Results

**Figure 5:**
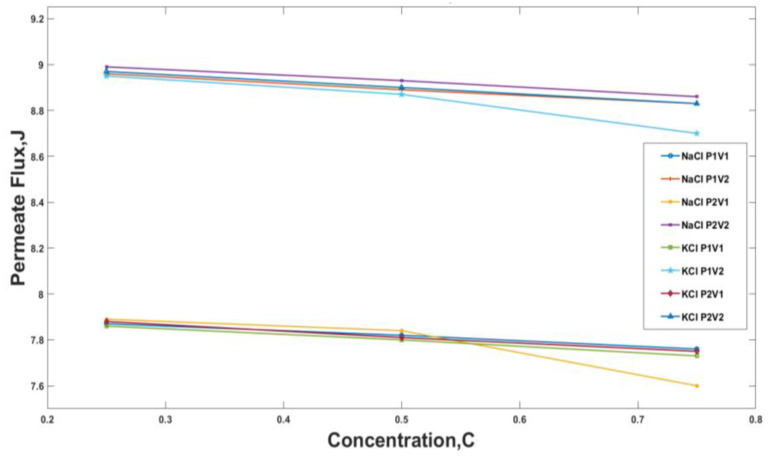
Permeate flux for temperatures 50 °C, i.e., 333 K against feed concentration for different Pressure and Velocity Values for NaCl and KCl where, P1 = 30 kPa, P2 = 20 kPa, V1 = 3.48 m/s, and V2 = 5.22 m/s.

At a temperature of 50 °C, i.e., 333 K, the change in permeate flux for NaCl and KCl was observed at different pressures and velocity combinations. The results have been plotted in Figure 5. It was observed that the increase in the concentration of salt in the solution decreased the permeate flux. For example, at operating conditions 30 kPa and 3.48 m/s for NaCl, an increase in salts concentration from 0.25 to 0.75 decreased the permeate flux by 1.4%; a similar trend was obtained for KCl. This decrease was observed because the vapor pressure, the driving force behind membrane distillation, is reduced as the ion’s concentration near the membrane increases. In the NaCl solution, at a vacuum pressure of 30 kPa, the change in velocity from 3.48 m/s to 5.22 m/s increased the permeate flux. For KCl, a constant velocity, for example 3.48 m/s, and an increase in pressure from 20 kPa to 30 kPa resulted in a 2% increase in permeate flux; however, the case for NaCl was slightly different, i.e., 1.4%. The difference in the permeate flux between NaCl and KCl is due to the difference of diffusivity between them. KCl has a higher diffusivity than NaCl due to its smaller ionic radius and lower hydration energy. This means that K^+^ and Cl^−^ ions can more easily pass through the membrane than Na^+^ and Cl^−^ ions, resulting in a higher permeate flux for the KCl solution compared to the NaCl solution. Additionally, the surface charge of the membrane can also play a role in determining the selectivity of the membrane towards different ions. Some membranes may have a higher affinity for K^+^ ions than Na^+^ ions, leading to a higher permeate flux for the KCl solution. At C1, maximum permeate flux in both cases, i.e., NaCl and KCl is reduced by about 15% at C3 because of reduced vapor pressure.

**Figure 6:**
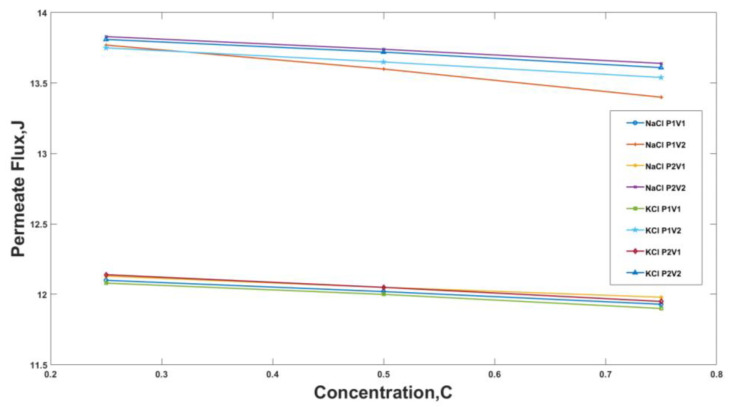
Permeate flux for temperatures 60 °C, i.e., 343 K against feed concentration for different Pressure and Velocity Values for NaCl and KCl where, P1 = 30 kPa, P2 = 20 kPa, V1 = 3.48 m/s and V2 = 5.22 m/s.

At a temperature of 60 °C, the change in permeate flux for NaCl and KCl solutions for various velocity and pressure combinations, the variation had been plotted in Figure 6. The increase and decrease trend for permeate flux for both the solutions, i.e., the NaCl and the KCl solution, were alike. For the NaCl solution at a temperature of 60 °C, it was observed that the increase in the concentration of salt in the solution decreased the permeate flux. Similarly, at a vacuum pressure of 30 kPa, the change in velocity from 3.48 m/s to 5.22 m/s increased the permeate flux. An increase in feed velocity should assist the desalination by increasing the turbulence and, therefore, increasing heat and mass transfer. At a constant velocity of 3.48 m/s and an increase in pressure from 30 kPa to 20 kPa, the trend was also similar, as a slight increase was observed in the permeate flux values. A 12% increase was observed for concentration C1 at change of pressure from 30 kPa to 20 kPa.

**Figure 7:**
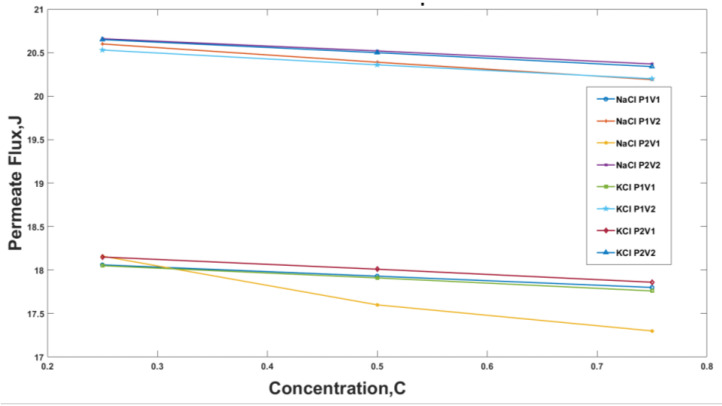
Permeate flux for temperatures 70 °C, i.e., 353 K against feed concentration for different Pressure and Velocity Values for NaCl and KCl where, P1 = 30 kPa, P2 = 20 kPa, V1 = 3.48 m/s and V2 = 5.22 m/s.

The permeate flux for the NaCl and the KCl solution at a temperature of 70 °C has been plotted in Figure 7. Similar to the above plots, it was noted that the increase in the concentration of salt in the solution decreases the permeate flux. For the NaCl solution, a decrease in permeate of 12.6% was observed for a constant velocity of 3.48 m/s and an increase in pressure from 30 kPa to 20 kPa. Additionally, it was observed that at constant pressure 30 kPa or 20 kPa and increase in velocity from 3.48 m/s to 5.22 m/s the permeate flux increased. The graphs trend for the KCl Solution were comparable to NaCl with a difference of a single case, i.e., 20 kPa, 3.48 m/s. The decrease in permeate flux for KCl for parameters 20 kPa, 3.48 m/s was linear while that of the NaCl solution was nonlinear.

The increase in temperature from 50 °C to 70 °C increases the permeate flux through the membrane. A 29% increase in the permeate flux was observed for 3.48 m/s, 30 kPa pressure, and 0.25 M concentration by increasing the temperature from 333 K to 353 for a sodium chloride solution. This increasing trend was common for salts, i.e., NaCl and KCl. This trend is observed due to an exponential rise in water vapor pressure on the feed side with a temperature rise which increases the permeate flux of the solution. For both the KCl and KCl solutions and concentration C1, the change in temperature from 50 °C to 60 °C resulted in a 53% increase in permeate flux, while for a shift from 60 °C to 70 °C, the recorded increase was 49%.

**Figure 8:**
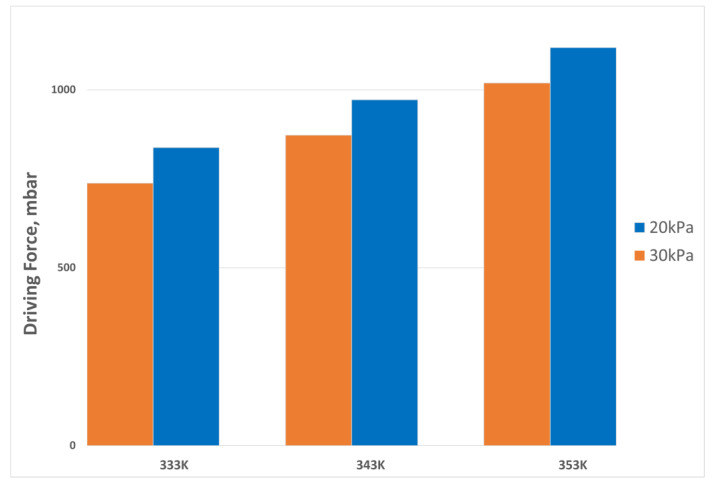
Process Driving force.

The process driving force, being a critical parameter in VMD, was calculated for different temperature and pressure values using Equation (1). The results are presented in a bar graph in Figure 8. The minimum driving force was observed at a temperature of 333 K and a pressure of 30 kPa, while the highest driving force was observed at a temperature of 353 K and a pressure of 20 kPa. The driving force effects the efficiency and productivity in VMD process. A higher driving force leads to faster separation rates and increased productivity, while a lower driving force reduces efficiency and productivity. The study’s findings on the relationship between driving force and temperature/pressure conditions have important implications for optimizing VMD processes. By selecting optimal operating conditions, the efficiency and productivity of VMD processes can be improved, making it useful in various separation processes such as desalination and wastewater treatment.

### 3.2. Results of Economic Analysis

**Figure 9:**
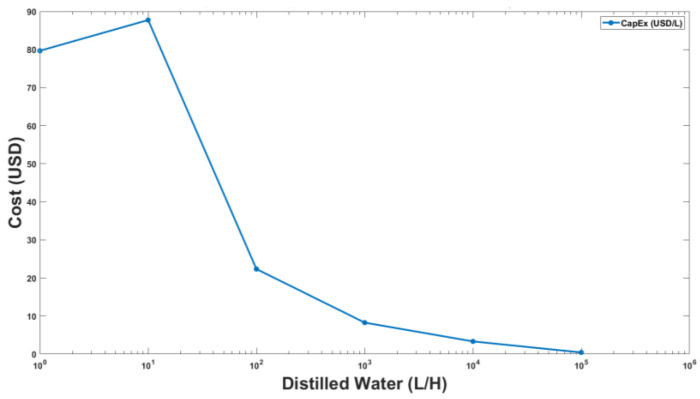
Capital Cost of VMD plant as a function of distilled water production capacity in Liters per hour (L/H).

For the lab scale model, the total cost incurred is described in Table 3. Since technical support and instrumentation were available in the lab, the installation and instrumentation costs of the unit is taken as 25% of the cost of the lab scale vacuum membrane distillation plant as recommended in the literature [36]. The maximum and minimum permeate flux for NaCl solution was recorded as 20.37 and 7.87 L/m^2^_._H and, for KCl, as 7.73 and 20.65 L/m^2^_._H, respectively, where the duration of the experiment was in hours. To determine the costs of the larger scale units capable of producing greater permeate flux, a market survey was conducted to estimate the prices for essential components, i.e., feed water pump and vacuum pump for the vacuum membrane distillation unit at different scales from a few liters of permeate flux to 100,000 Liters/hour. Installation and commissioning cost of the distillation unit along with the miscellaneous cost was calculated. While estimating the price, researchers [37] have considered 25% of total plant cost as installation cost, while, in this research, a nonlinear cost function for installation was considered that is inversely proportional to the size of the plant such that the smallest units have 60%, whereas 20% for the largest unit is considered. The capital cost for various capacities of vacuum membrane distillation plants obtained is shown in Figure 9.

The focus of techno economic analysis is to provide the starting point for potential investors in VMD distillation. Some of the challenges in moving from lab scale to industrial scale of VMD process include high energy consumption, fouling, scaling, and pore wetting [35]. The errors in cots estimation will be dependent on diverse economic factors such as currency exchange rate fluctuation, global supply chain disruptions, fluctuation in the costs of energy, and local variations due to variable inflation in different parts of world. This study, therefore, serves to provide an indicative cost to potential investors.

**Figure 10:**
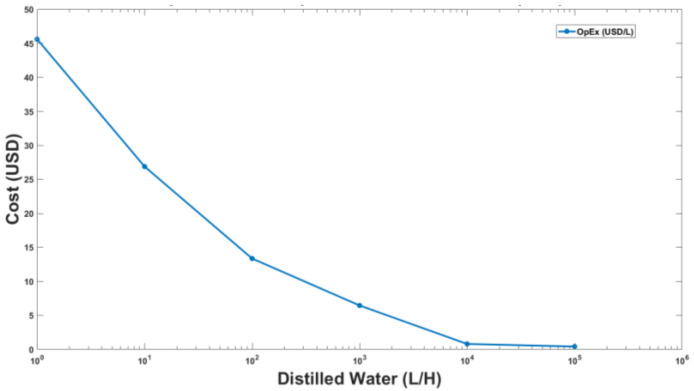
Operating cost of VMD plant in (USD/L) as a function of production capacity.

The capital and operating expenses for the upscaled distillation plant were calculated in USD/L by considering the optimal power consumption of a feed water pump and vacuum pump along with nominal electricity charges and are shown in Figure 10. The nominal electricity charges were taken from the local electricity provider [37]. The optimal power consumption was calculated by using the rated power of the pumps with their operating hours in a day. Miscellaneous plant operating and maintenance costs were also considered as a range of 10% to 22% of base operating cost in proportion to the scale of the plant.

**Figure 11:**
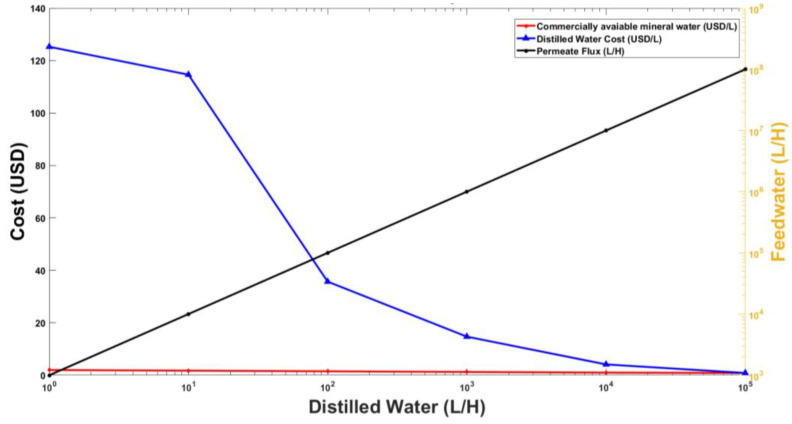
Breakeven for VMD plant of various scales.

The suggested desalination model achieves its breakeven point against the commercially available mineral water when distilled water production of 100,000 L/H is shown in Figure 11. Figure 11 takes into account both capital and operational expenses and feed water flow rate required to produce varying quantities of distilled water. At lab scale, the unit cost of distilled water is very high, which can be brought down significantly after increasing production volume. The graph indicates a breakeven for distilled water production from a larger version of the lab scale VMD unit at a production capacity of 100,000 L per hour. To achieve this rate of permeate flux in the VMD Plant, it will be taking in approximately 100 million liters of sea water per hour as indicated by the black line in Figure 10 above.

### 3.3. Full-Scale Plant Model

**Figure 12:**
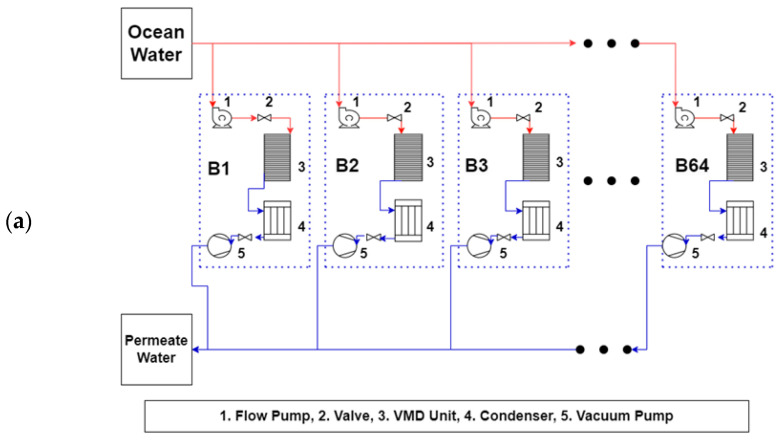

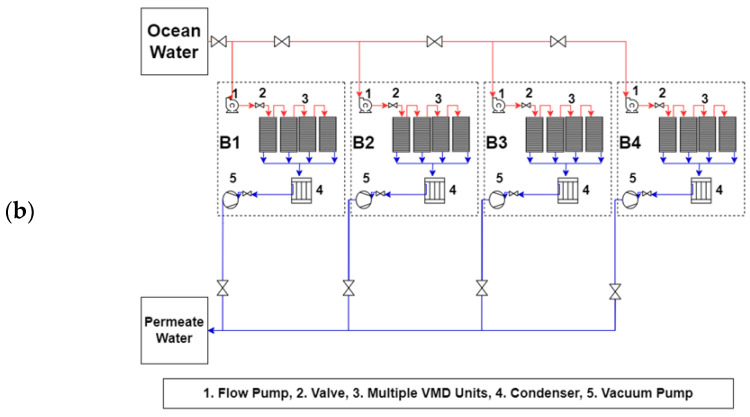
Proposed layout of VMD plants range of integration: (**a**) 64 VMD units and (**b**) 4 multi-effect vacuum membrane units.

For the commercial VMD processes, plant layouts are suggested in Figure 12a,b, each with a different number of pumps and membrane units. The first approach used 64 small units equipped with 0.5 horsepower permeate and vacuum pumps, while the second approach employed 4 multi-effect vacuum membrane units with a 128 horsepower permeate and vacuum pump each. The first approach has the advantage of availability of pumps, but also presents an additional challenge of a complex maintenance procedure due to the large number of units. The second approach represents a reduced unit count and simpler maintenance regime, but with the disadvantage of the limited availability and high cost of the feedwater and vacuum pumps. There are a wide range of options between these two methods depending on factors such as the specific requirements of the industrial facility and resource availability. These results stress the importance of carefully evaluating the trade-offs between different approaches to optimize the efficiency and cost-effectiveness of the industrial vacuum membrane desalination process.

## 4. Discussion

Seawater desalination is a process that involves the removal of salts from seawater to make it potable. One of the technologies that can be used for desalination is Vacuum Membrane Distillation (VMD). This current study focuses on the cost analysis of a VMD setup for removing salts from water using a Polytetrafluoroethylene (PTFE) membrane with 250 mm × 200 mm dimensions and 165 µm thickness.

The study conducted a total of 72 experiments with variations in parameters such as velocity, pressure, concentration, and temperature. The experiments were conducted with NaCl and KCl solutions, with 36 experiments for each solution. The results from these experiments were plotted in Figure 5, Figure 6 and Figure 7.

It was observed that the increase in concentration of salt in the solution decreased the permeate flux, which is the amount of water that passes through the membrane. For example, at operating conditions of 30 kPa and 3.48 m/s for NaCl, an increase in salts concentration from 0.25 to 0.75 decreased the permeate flux by 1.4%. This decrease was observed because the vapor pressure, the driving force behind membrane distillation, is reduced as the ion’s concentration near the membrane increases.

It was also observed that an increase in temperature from 50 °C to 70 °C increases the permeate flux through the membrane. A 29% increase in the flowrate was observed for a 10 L/min flow rate, 30 kPa pressure and 0.25 M concentration when the temperature was increased from 50 °C to 70 °C for a sodium chloride solution. This increasing trend was common for both NaCl and KCl solutions. This trend is observed due to an exponential rise in water vapor pressure on the feed side with a temperature rise which increases the permeate flux of the solution.

The cost analysis of the desalination process discussed in this paper is based on various factors such as capital costs, operation and maintenance costs, installation costs, and human resource costs. The capital and operating expenses were estimated for the up-scale model using the equipment factor estimate method. This method involves taking the operational and capital cost and then adding the human resource and miscellaneous cost factors. The calculations were carried out for a range of investments from lab scale setup with 1 L/h capacity to an upscaled industrial model of 1,000,000 L/h capacity. The important cost analysis parameter included was the breakeven point for the proposed model in comparison with commercially available bottled water. A significant percentage of the cost of desalination setups consisted of the operating costs which included the electrical and maintenance cost of the pumps. The pumps capital and operating costs for different proposed scales of VMD plants were calculated with the help of local and globally available pumps. The human resource and miscellaneous costs were different for lab and industrial setups, with the percentage of human resource cost for lab scale greater than that of industrial level while miscellaneous costs greater in case of an upscaled model. While reverse osmosis remains one of the most widely used desalination methods, the present study identifies the scale at which VMD should be deployed to be commercially competitive.

## 5. Conclusions

The VMD technique was used for the desalination of seawater at lab scale using dissolved NaCl & KCl solution as feed water at various operating conditions of temperature and pressure. The parameters selected were velocity (3.48 m/s, 5.22 m/s), pressure (30 kPa, 20 kPa), and temperature (50 °C, 60 °C, and 70 °C) for the experimentations. A total of 72 experiments were performed for this study which yielded significant results. For the NaCl solution, the change in temperature from 50 °C to 70 °C increased the permeate flux, while change in velocity and pressure contributed to both an increase and decrease in flux through the membrane. At 30 kPa & 3.48 kPa, increasing the salt content from 0.25 to 0.75 concentration, reduced the permeate flow by 1.4%. This pattern is seen because the vapor pressure, which drives membrane distillation, decreases as ion concentration near the membrane rises. However, the change in velocity from 3.48 m/s to 5.22 m/s increased the permeate flow at a vacuum pressure of 30 kPa. The rise in concentration C1 recorded from 30 kPa to 20 kPa was 12%. The resulting trend for the KCl solution was similar to the NaCl with slight variations in the increase and decrease of permeate flux. At a temperature of 50 °C, it was observed that increased concentration from 0.25 to 0.75 significantly reduced the permeate flow. The reduction in permeate flow was nonlinear for a given pressure of 30 kPa and a velocity of 5.22 m/s but linear for all other variables. Additionally, it was observed that, with an increase in temperature from 60 to 70 °C, the permeate flux for concentration 0.25 was 49% for all the combinations of pressure and velocity. Subsequently, an economic analysis was carried out based on experimental results and coupled with a market survey to estimate the cost of a large scale VMD Plant. The study shows that by using the VMD technique, the goal of removing salts from seawater could be achieved with the help of a large-scale vacuum distillation plant providing 100,000 L/H of distilled water based on the current experimental setup. The process is suitable for commercialization if the distilled water output is 100,000 L/H. This is because at this rate the industrial unit cost reaches breakeven with the commercial water bottle cost.

## Figures and Tables

**Table 1:** Specifications of Polytetrafluoroethylene (PTFE).

Property	Specification
Material	PTFE
Dimensions (Length × Width)	250 mm × 200 mm
Thickness	165 µm
Effective Area	0.0336 m^2^
Porosity	70–75%
Pore size	0.2 µm

**Table 2:** Parameters used in our experimentations.

Parameters	Variation	Value
Concentration, M	C1	0.25
	C2	0.5
	C3	0.75
Pressure, kPa	P1	30
	P2	20
Velocity, m/s	V1	3.48
	V2	5.22
Temperature, K	T1	333
	T2	343
	T3	353

**Table 3:** Cost estimation of lab scale unit.

	Component	Cost ($)
Capital Cost	Membrane	$36/m^2^
	Feedwater Pump	25 $
	Vacuum Pump	26 $
Installation cost of VMD	Installation Cost	25% of Total Equipment Cost
	Instrumentation and Control Cost	25% of Total Equipment Cost

## Data Availability

Not applicable.
